# Composition of homegarden plants and cultural use in an indigenous community in Northwest Ethiopia

**DOI:** 10.1186/s13002-022-00545-5

**Published:** 2022-06-20

**Authors:** Metsehet Yinebeb, Ermias Lulekal, Tamrat Bekele

**Affiliations:** 1 Plant Biology and Biodiversity Management, College of Natural Sciences, Addis Ababa University, P.O. Box 1176, Addis Ababa, Ethiopia; 2 Biology Department, Kotebe University of Education, Addis Ababa, Ethiopia

**Keywords:** Conservation, Cultural importance, Cultural value, Gozamin District, Use diversity

## Abstract

**Background:**

Homegardens in Northern Ethiopia received little investigation into the diversity of plants and no study and recording in the Gozamin District. This study was used to fill the gap in how cultural use and cultural importance conserve species diversity in homegardens in the different agroclimatic zones in northwestern Ethiopia.

**Methods:**

The study district and 12 kebeles were chosen using multistage and stratified random selection procedures based on traditional agroclimatic zones in the Gozamin District, Northwest Ethiopia, respectively. The number of plots chosen in each homegarden was determined by the homegarden's size, which ranges from 0.015 to 0.5 ha. These data were gathered by putting plots with a distance gradient from home (size: 10 × 10 m each). A semi-structured interview and complete plant inventory were conducted to document the informant's knowledge of plant species. Sørensen’s similarity indices and Shannon–Wiener diversity indices were used to compare the similarity of sites and three agroclimatic zones, respectively. Direct matrix ranking, cultural importance (CI), the relative frequency of citation, and cultural value were used in quantitative analysis to compare the most common multipurpose plants.

**Results:**

A total of 238 culturally important plant species from 81 families were identified. The Kruskal–Wallis test showed that there was a significant difference among the three agroclimatic zones species diversity (*H* = 103.4, Hc = 111.2, *p* < 0.05). Of the total plant species recorded, 59% were reported to be utilized for environmental uses, 35% were food crops, and 35% were medicinal plant species. The same was true for the three agroclimatic zones; food and medicinal uses were the first and second most important use categories, respectively. The similarity index for 64% of the sites investigated was less than 0.5. *Cordia africana* (FC = 125) was the most culturally significant species with a value of 2.23 on the CI index.

**Conclusion:**

Homegardens are multifunctional systems. The presence of different agroclimatic zones, cultural uses, cultural importance, and cultural value of the species are central to conserving plant species in the area. As the size of the garden increases, so does the diversity of species and uses. Our findings suggest that conservation strategies should take into account the links between plant composition and cultural importance.

## Background

Homegarden can be defined as planting multipurpose trees along with crops and livestock around the homestead or preparing vegetable planting and arrangement of quality vegetable seeds to extend the year-round supply of nutrient-rich nourishment inside a family [1]. The wide range of products cultivated in homegardens from trees, shrubs, and herbaceous plants provide variety to rural households' diets [2] and also serve as important sources of cash income through the sale of surplus produce and cash crops [3]. In agreement with Landon-Lane [1], homegardens, including trees, shrubs, and herbaceous plants that grow in or near a homestead or home compound, are planted and managed by family members, and the goods and services are largely for household consumption and ornamental value. Homegarden production, according to Kumar and Nair [4], is mostly supplementary to staple food production and primarily focuses on vegetables, fruits, and condiments. It tends to have a few trees that can be utilized for the long-term generation and deal for benefit. Homegardens are also critical territories for therapeutic plants all over the world [5].

Around the world, homegardens are a community’s most versatile and available arrive resources and critical components in diminishing vulnerability and guaranteeing nourishment security [6]. Year-round food production, decreased risks of production failure owing to high species diversity, increased resource productivity over time, expansion of the amount and quality of labor employed in the farm, output flexibility, and alternative production are features of homegardens [7]. Concurring to Das and Das [8], homegarden frameworks give an extra nourishment supply and cash pay for the individuals. Tropical homegardens are also generally regarded as sustainable production systems [9, 10]. The choice of plant species, their arrangement, and management vary between and within tropical homegardens in the same community [11]. Better market access, as well as greater public promotion of homegardens, encourages their adoption, but only in more water-abundant ecologies [12]. According to Galluzzi et al. [13], homegardens, whether in rural or in urban settings, are multifunctional, allowing them to provide a variety of advantages to ecosystems and people.

Homegarden programs are these days more broadly executed in Africa [14, 15]. The expansive concentration of the valuable plants found in Ethiopia is found in homegardens [16]. Agreeing with Eyasu et al. [17], in Northern Ethiopia gardening complements the natural forest in terms of biodiversity conservation and aids in the prevention of the extinction of woody species in the natural environment. Amberber et al. [18] and Kewessa [19] also reported that homegardens are in situ conservation sites of biodiversity in different parts of Ethiopia. Homegardens are fundamentally distinct from large-scale agricultural systems. The size of homegardens in Ethiopia ranges from 0.004 to 0.05 ha [20]. Farmers' seed and crop management decisions, as well as adjacent modes of living, are shaped by cultural traditions [12]. At the same time, homegardens are important social and cultural sites where agricultural expertise is passed along [13]. Homegardens also have great social and cultural values in several regions of the world [21]. They also meet social necessities and give environmental administration besides serving additionally as a source of income [22]. Thus, researching home gardens was viewed as an efficient technique to learn the biodiversity-related local knowledge and culture of native people [23].

In Northern Ethiopia, forest loss is intensifying [24] and homegardens are used as sites of in situ conservation of plants traditionally grown for cultural use [16, 17]. The majority of homegarden plant composition takes place in the country's South and Southwest with different agroecology and composition when compared to that of the north of Ethiopia [25–27]. Different types of homegardens were recognized according to Nair [28] based on differences in size (area), shape, layout, zoning scheme, species composition, management aims, dominating plant species, and urbanization level. In northwestern Ethiopia, little research has been done on the diversity of homegarden plants [29, 30]. Therefore, this article examines how cultural use is used for maintaining plant species diversity in homegardens in northwestern Ethiopia, as there is no record of the diversity and cultural use of homegarden plants in the current area. Here, it has been hypothesized that the composition of plant species in homegardens in the Gozamin District of Northwest Ethiopia depends on differences in agroecological zones (*Dega*, *Kolla*, *Woina Dega*) affecting species presence, and variations in the cultural use, cultural value, and cultural importance related to the species available in the three agroecological zones (*Dega*, *Kolla*, and *Woina Dega*). This study was conducted with the aim of (1) documenting the composition of culturally useful plant species in homegardens, (2) showing the diversity and use differences in the three agroecological zones, and (3) affirming the cultural use, cultural value, and cultural importance of plant species at homegardens in the Gozamin District of northwestern Ethiopia.

## Materials and methods

### Study area

**Fig. 1:**
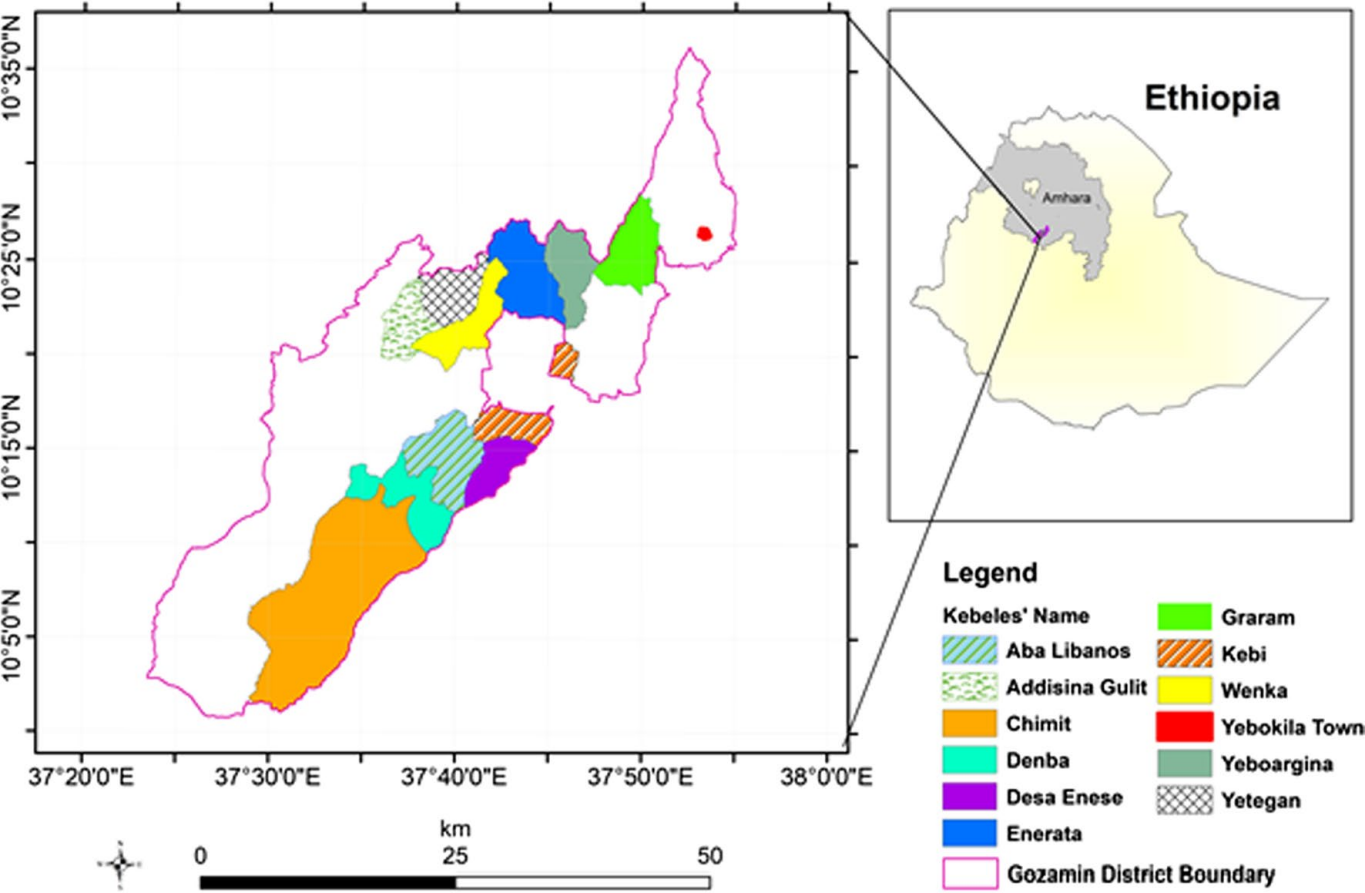
Map of Ethiopia showing the Amhara region and the study district that inhabits an indigenous community

The research was carried out in Northwest Ethiopia's Gozamin District, east Gojjam. It is one of the 20 woredas of Amhara National Regional State's East Gojjam zone [31]. The Gozamin District is located 300 km north of Addis Ababa, Ethiopia's capital, and 260 km southeast of Bahir Dar, the Amhara Region's major administrative headquarter. Gozamin is bounded on the east by Aneded and Debay Tilatgen districts, on the west by Machakle and Debre Elias districts, on the north by Senan District, and on the south by Baso Liben District and Oromia National Regional State (Fig. 1). Agriculture is the most prevalent source of income in Gozamin District. The district encompasses 121,781 ha in total, with 50,084 ha of agricultural land, 18,966 ha of pasture land, 22,225 ha of forest, and 30,506 ha of other lands [31]. According to the traditional agroclimatic zones classification, 1% of the district belongs to *Wurch*, 9% *Dega*, 79% *Woina Dega*, and 11% *Kolla*, whereas the elevation of the district ranges between 800 and 3748 m a.s.l. The average annual rainfall in the area is from 1000 to 1510 mm, while the average annual temperature ranges from 8.5 to 30 °C [31]. Gozamin District has a total population of 170,690, with 85,220 men and 85,470 women [32]. In terms of human health, the city has 6 health centers, 27 health posts, 3 private clinics, and 1 private pharmacy (drug store).

### Sampling method

The study was designed following a scoping survey which was conducted in the east Gojjam zone in June 2019, and preliminary information was gathered on agro-climatic zones, landscapes, and agricultural methods through a field survey walking in the area and from the zone's agricultural offices. The Gozamin District was chosen from the East Gojjam zone after a multistage sampling procedure. First, a total of 12 kebeles (the smallest administrative units in the Ethiopian administrative system) were identified from the total 25 kebeles available in the study district based on a stratified random sampling procedure focusing on agroclimatic zones (i.e., *Dega*, *Kolla*, and *Woina Dega*) and the extent of homegarden agroforestry practices. Second, the kebeles were stratified into three groups based on proper agroecology, in that four kebeles (Chimit, Denba, Desa Enese, and Aba Libanos) were parts of *Kolla*, and six kebeles (Addisenagulit, Kebi, Wenka, Yetegan, Yeboargina, and Enerata) belonged to *Woina Dega*, and the other two kebeles (Graram and Yebokla) belonged to *Dega* (Fig. 1). Third, using Cochran's [33] formula, we calculated the number of houses to be included in the study from the 12 kebeles.

$$n = \frac{{n_{0} }}{{1 + \frac{{\left( {n_{0} - 1} \right)}}{N}}},$$

where *n* = number of households sampled from all kebeles, no is the sample size and it is expressed as $n_{0} = \frac{{Z^{2} pq}}{{e^{2} }}$, *z* = the selected critical value of desired confidence level (1.96); *p* is the estimated proportion of an attribute that is present in the population, *q* = 1 − *p* and *e* = the desired level of precision (0.05).

Finally, using proportions, 30 households were chosen from each of the 11 kebeles, whereas 27 households were chosen from a single kebele. The survey included 357 homes or homegarden owners out of a total of 5056 homegarden owners in the 12 kebeles. There were 102 women and 255 men among the 357 homegarden owners interviewed, ranging in age from 20 to 86 years (Appendix 1). We chose 24 key informants (two knowledgeable people from each kebele).

### Data collection

**Fig. 2:**
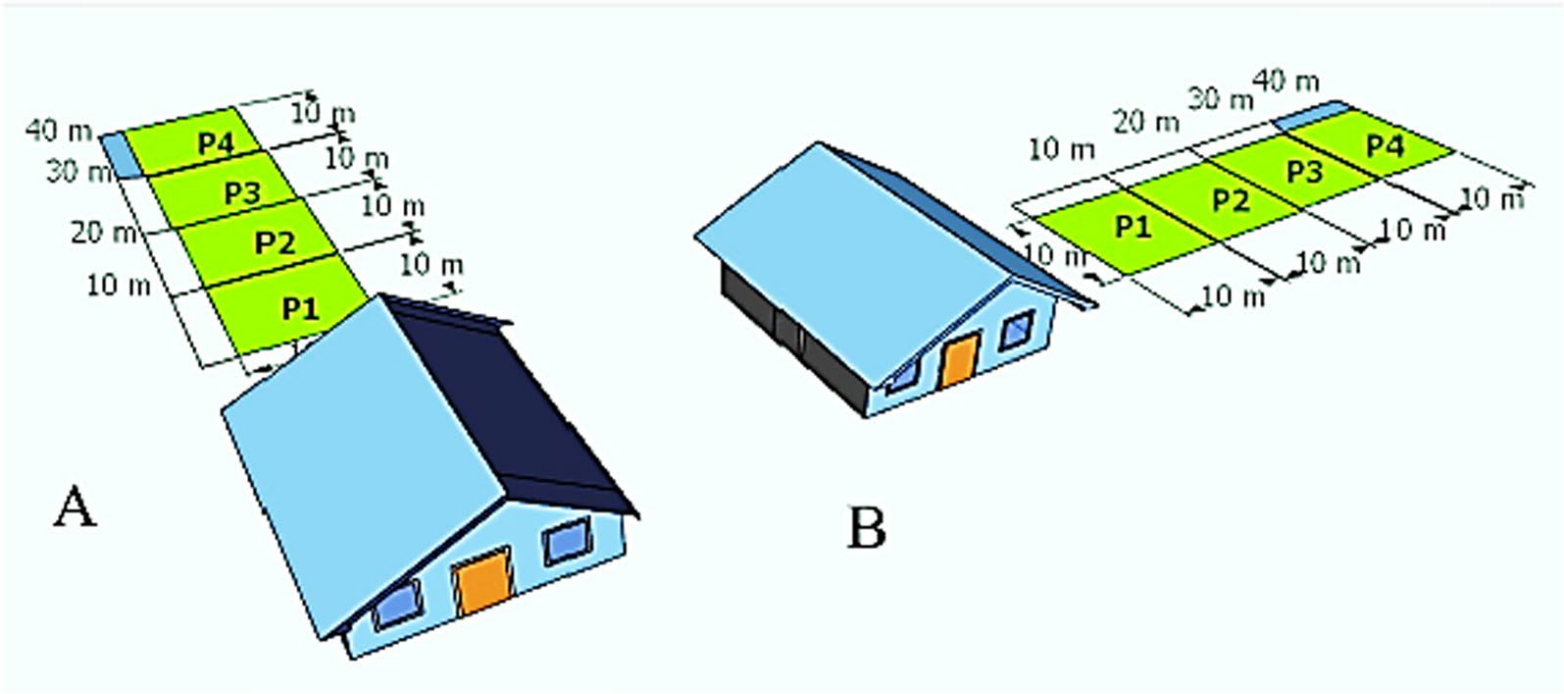
Map illustrating the homegarden plot sampling technique (note that p means plot). **A** Homegardens at the back of the home and **B** homegarden at the side of the home. *Map by sketch up pro 2021 version 21.1.279

The data on culturally important plant (herbaceous and woody) species composition, abundance, and the farmers' use of these plants were collected from homegardens from March to April 2020 and July to August 2021. These data were collected by laying out plots with a distance gradient from home (size: 10 × 10 m each). The number of plots chosen in each homegarden was determined by the size of the homegardens, which ranged from 0.015 to 0.5 ha, and the minimum number was one plot and the maximum number was four plots (Fig. 2).

Herbaceous species data were collected from subplots (every 1 × 1 m in size) located at the four corners and center of each of the main plots. For data collection from 357 homegardens, 898 main plots and 4490 subplots were used. A complete plant inventory and a semi-structured questionnaire were used to gather the cultural uses of the plants.

### Plant specimen collections and identification

Homegarden plant species in each plot were recorded, collected, pressed, dried, and mounted. The voucher specimens were identified at a species level using validated herbarium samples and published flora volumes of Ethiopia and Eritrea. The botanical identities of voucher specimens were confirmed by experts at the National Herbarium, Addis Ababa University. All plant specimens were deposited at Addis Ababa University's National Herbarium, Addis Ababa, Ethiopia.

### Data analysis

#### Descriptive statistics

Homegarden data were analyzed and summarized using different descriptive statistical methods (percentages, graphs, charts, tables) using Excel 2016 and PAST 4.03 [34].

#### Similarity indices

The Sørensen similarity coefficient was utilized to compare the similarity within the species composition across homegardens since it gives more weight to the species that are common within the kebeles instead of those that are present in either kebele. The Sørensen similarity index (*S*_s_) was calculated by using:

$$S_{{\text{s}}} = \frac{2a}{{2a + b + c}}$$

where *a* is the number of species common to both samples, *b* is the number of species in sample 1, and *c* is the number of species in sample 2 [35].

#### Shannon–Wiener diversity indices

The diversity of plant species was analyzed by using Shannon–Wiener diversity indices to calculate the diversity of homegarden plants in three agroclimatic zones as described by Kent and Coker [36]. The Shannon–Wiener diversity index was calculated as

$$H = - \sum\nolimits_{i = 1}^{s} {\text{Pi }}\ln \, \left({\text{Pi}}\right)$$

where *H* = Shannon–Wiener diversity index of the species, *s* = number of species recorded in each homegarden, and pi = proportion of the abundance of each species from the total abundance of plant species recorded in each homegarden. Using PAST software version 4.03, the Kruskal–Wallis test and Dunn's post hoc were used to determine whether there are significant differences in species diversity among different agroclimatic zones.

### Direct matrix ranking

The direct matrix ranking approach was used to rank culturally important multipurpose species which are under higher human pressure, including the threats they faced. Several aspects of plants were considered, including their usage as food, fodder, environmental purposes, medicine, materials, fuel, and social uses. Based on information obtained from informants, fifteen mostly useful multifunctional tree species were chosen, and seven use diversities of these plants were listed for 24 key informants to assign functional values to each species [37]. The functional values (5 = best, 4 = very good, 3 = good, 2 = least used, 0 = not used) were allocated by each selected key informant. Then, the functional values for each species were summarized and ranked.

### Relative frequency of citation (RFC)

The proportion of each type of use and other indices were calculated using the frequency of citation (FC) method. The FC was determined as the total of informants who mentioned a certain species' usage (in this case, home gardeners with useful species in their gardens). The relevance of each culturally relevant species in the research area was determined using the relative frequency of citation (RFC) method [38]. The RFC values were obtained using the following formula.

$${\text{RFCs}} = \frac{{{\text{FCs}}}}{N}$$

where FCs is the frequency of citation (the number of home gardeners that have species in their garden), and *N* is the total number of informants in the survey. A high RFC number for a species suggests that it is used often and by a large proportion of the study's informants.

### Cultural importance (CI)

The cultural important index (CI) was developed to indicate the prevalence and diversity of use of each culturally important species. The value of CI was computed by summing the proportion of informants who cited each of the usage categories for a culturally significant plant in their garden [38]. Using the following formula:

$${\text{CI}} = \mathop \sum \limits_{u = 1}^{{{\text{Nc}}}} \mathop \sum \limits_{i = 1}^{N} \frac{{{\text{URui}}}}{N}$$

where UR represents the total number of informants and NC represents the number of use reports for available species in their garden, the total number of use reports for each use category of a specific culturally important species was URui, and the total number of informants was *N*, and the total number of use categories was NC.

### Cultural value index

The cultural value index (CV) was derived as the sum of three components using the following formula [38, 39]:

$${\text{CVs}} = \left[ {\frac{{{\text{NUs}}}}{{{\text{NC}}}}} \right] \times \left[ {\frac{{{\text{FCs}}}}{N}} \right] \times \left[ {\mathop \sum \limits_{u = 1}^{{{\text{Nc}}}} \mathop \sum \limits_{i = 1}^{N} \frac{{{\text{URui}}}}{N}} \right]$$

where the first is the number of cultural use categories for the culturally important species (NUs) divided by the total number of all cultural use categories (NC). The second component is the species' relative frequency of citation (RFC). Third is the cultural importance index of the given, culturally significant species (CI).

## Results

### Composition of homegarden plants

**Fig. 3:**
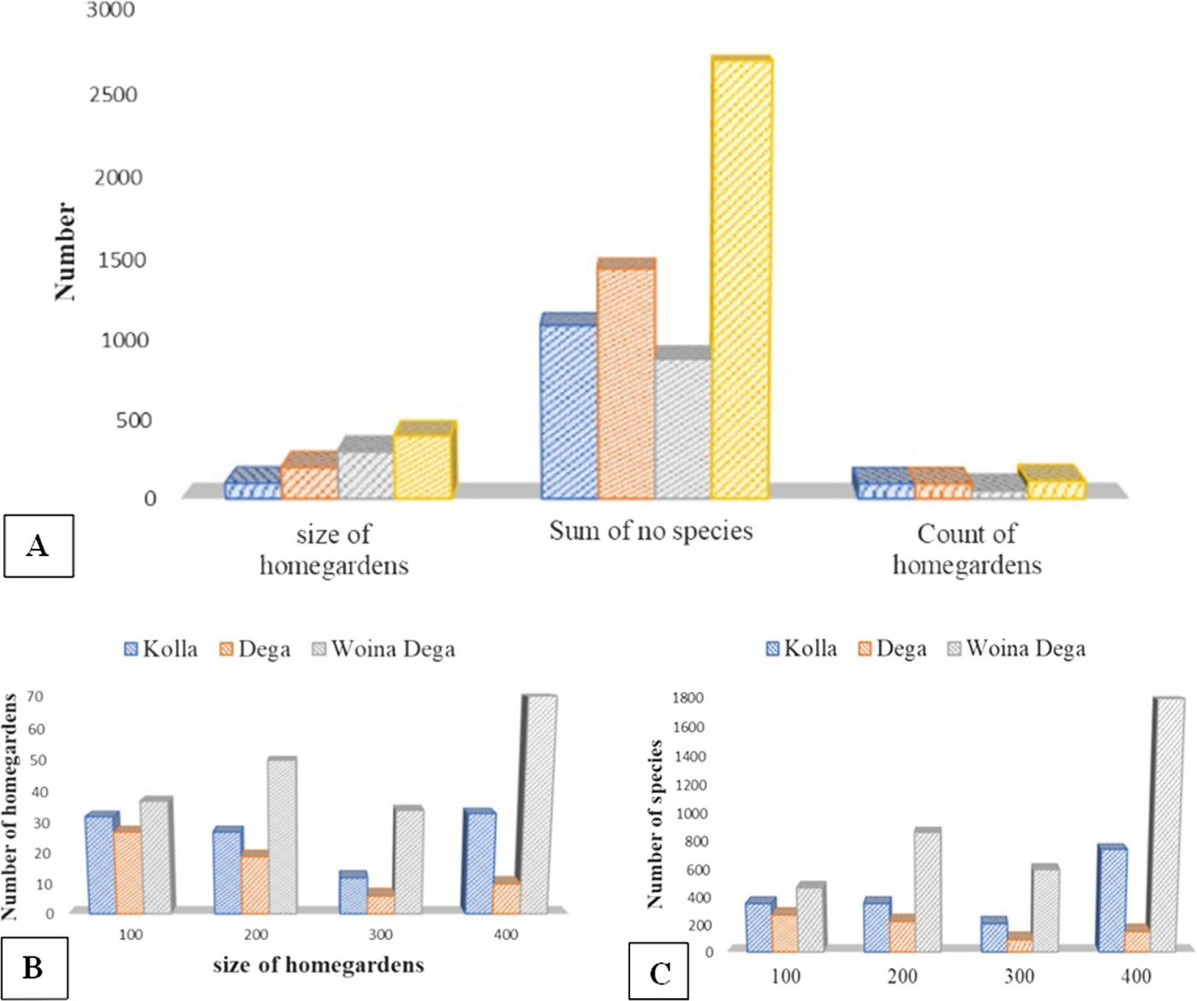
Size of homegardens and number of species in the study area (**A**) and in three agroclimatic zones (**B**, **C**)

The size of homegardens used for plantations in the study area ranged from 0.015 to 0.5 ha. The total area sampled (plots were taken) was 8.98 ha for the 357 homegardens. Of the overall 357 homegardens species, a total of 238 species were found culturally important belonging to 81 different botanical families. The number of species increases as the size of homegardens increases, but decreases in homegardens with three plots, as shown in Fig. 3, and the same is true in the *Dega*, *Woina Dega*, and *Kolla* agroclimatic zones. Of these collected homegarden plants, 128 plant species were cultivated and 110 species were wild. Wenka kebele had the highest homegarden species (133 species belonging to 61 families), while Graram kebele had the least, with 59 species and 30 families (Table 1).

**Table 1:** Number of plant species in sampled kebeles

Study sites (Kebeles)	Agroecology part taken	Number of botanical families	Number of species (richness)
Aba Libanos	Woina Dega partial Kolla	50	116
Addisenagulit	Woina Dega	43	94
Chimit	Kolla	33	61
Denba	Kolla	43	88
Desa Enese	Kolla	40	77
Enerata	Woina Dega (partial Dega)	41	94
Graram	Dega	30	59
Kebi	Woina Dega	30	63
Wenka	Woina Dega	61	133
Yeboargina	Woina Dega	40	76
Yebokla	Dega	35	67
Yetegan	Woina Dega	42	89
Total		81	238

**Fig. 4:**
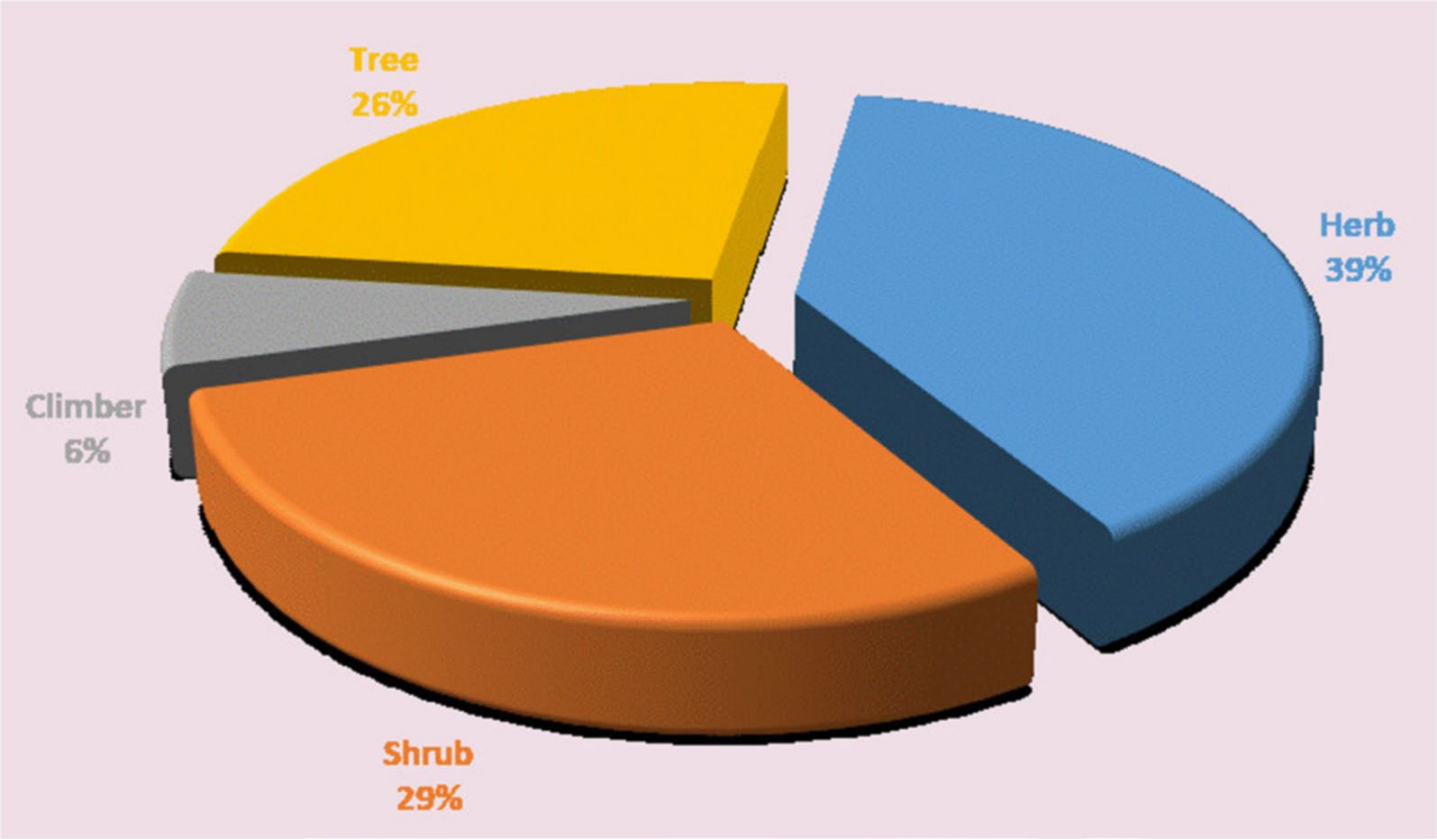
Habits of important homegarden plant species recorded in the study area

Among the 238 plant species, 15 (6.3%) were climbers, 93 (39%) were herbs, 69 (29%) were shrubs, and the other 61 (25.6%) were trees (Fig. 4). Moreover, Poaceae had the highest number of species recorded with 22 (9.2%) species, followed by Fabaceae with 21 (8.8%) plant species and Asteraceae with 15 (6.3%) species. Most home garden species were perennials (83%), while annuals made up the least (17%).

### Diversity of homegarden plant species in three agroclimatic zones

In this study, *Woina Dega* agroclimatic zone had the highest species diversity (203 species), than *Kolla* (130 species) and *Dega* (88 species). Evenness was also highest in *Woina Dega* and lowest in *Dega* agroclimatic zones. However, the dominance value was highest in *Dega* agroclimatic zone and lowest in *Woina Dega* (Table 2).

**Table 2:** Homegarden plant species diversity in three agroclimatic zones of Gozamin District

	*Dega*	*Kolla*	*Woina Dega*
Taxa_S	88	130	203
Individuals	730	1542	3717
Dominance_D	0.03	0.03	0.02
Simpson_1-D	0.97	0.97	0.98
Shannon_H	3.9	4.08	4.48
Evenness_e^H/S	0.57	0.45	0.43
Brillouin	3.71	3.93	4.38
Menhinick	3.26	3.31	3.33
Margalef	13.2	17.57	24.57
Equitability_J	0.87	0.84	0.84
Fisher_alpha	26.16	33.85	46.12
Berger-Parker	0.07	0.06	0.05
Chao-1	98.69	146.4	239.4

Before the data analysis was conducted, the assumption of the normal distribution was checked using the Shapiro–Wilk’s normality test. Here, the *p* value is less than 0.05 in all agroclimatic zones (Appendix 2). Hence, we conducted the Kruskal–Wallis test and the test for equal medians showed that there was a significant difference among the three agroclimatic zone species diversity (*H* = 103.4, Hc = 111.2, *p* < 0.05). As indicated in Table 3, Dunn's post hoc comparison also revealed a significant difference within three agroclimatic zones.

**Table 3:** Dunn’s post hoc comparison among the three agroclimatic zones

	Dega	Kolla	Woina Dega
Dega		0.0004211	3.329E−25
Kolla	0.0004211		7.627E−12
Woina Dega	3.329E−25	7.627E−12	

### Cultural use classification of collected plant species

**Fig. 5:**
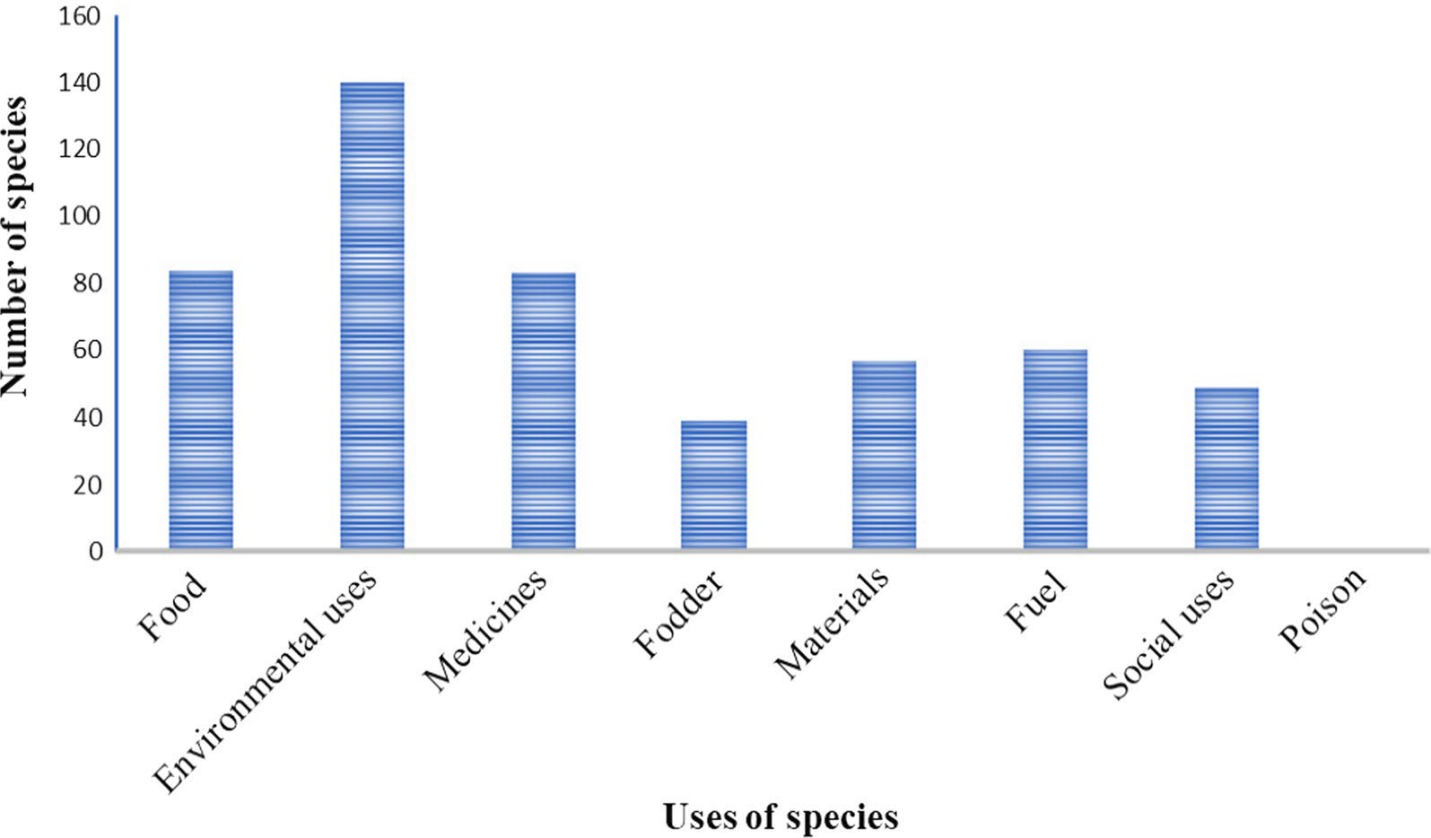
Importance of homegarden plant species in the study areas

**Fig. 6:**
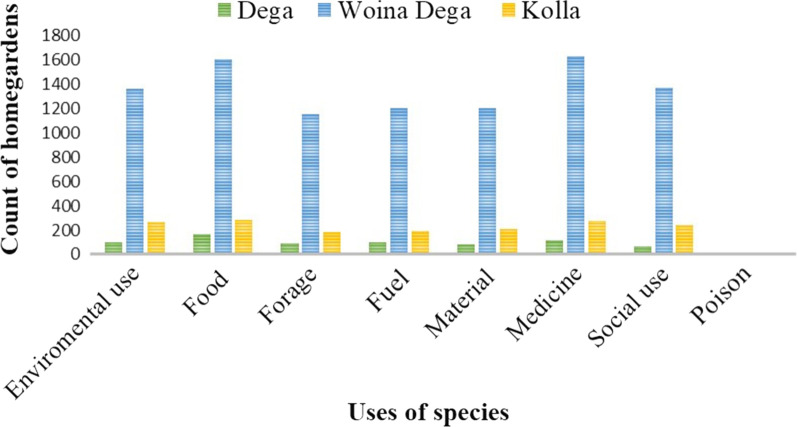
Uses of plants in different agroclimatic zones in Gozamin District

**Fig. 7:**
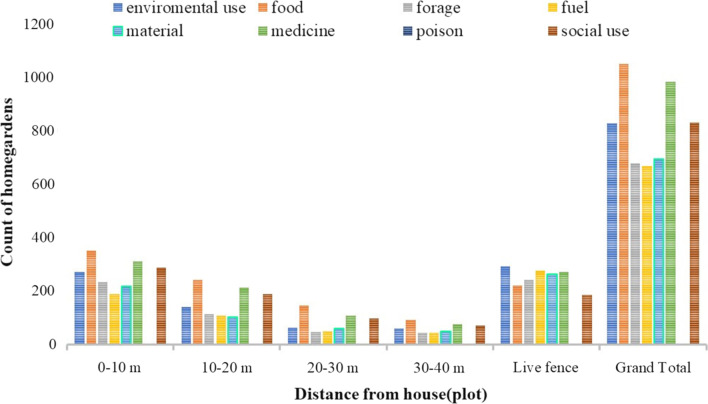
Plant use diversity in the different distances and live fence

Based on Cook [40], the collected 238 culturally important plant species were classified into eight use categories. Of the total plant species recorded, 140 (59%) were reported to be utilized for environmental uses, 84 (35%) food crops, 83 (35%) medicinal plant species, 39 (16.4%) fodder species, 57 (24%) material use plants, 60 (25%) fuelwood, 49 (20.6%) social use plants, and 1 (0.42%) poisonous plant (Fig. 5). Of the cultural uses of plants in the homegarden, the first use of plants recorded (frequented) in the homegarden was food (18.3%), medicinal value was the second (17%), and social use (14.5%) was ranked third (Figs. 6, 7).

### Food crops

A total of 84 (35%) food crops used as sources of fruit, vegetables, cereals, beverages, flavorings, spices, and gums were identified in the study area. An expansive number of species that belonged to food were Poaceae (8 species), Rutaceae (7 species), Fabaceae (6 species), Moraceae and Rosaceae (5 species each), Brassicaceae, Lamiaceae, and Solanaceae (4 species each), Alliaceae, Apiaceae, Asteraceae, Musaceae, Myrtaceae (3 species each), Anacardiaceae and Cupressaceae (2 species each), and the other 23 families had single species representation. Of these food crops, fruit plants were represented by 38 (45%) species. Numerous fruits that belonged to Rutaceae (6 species), Rosaceae, and Moraceae had 5 species each. The fruit plant species Kok (*Prunus persica*) and Duba (*Cucurbita pepo*) were found in all kebeles. The most commonly utilized flavors were *Rhamnus prinoides*, and the common vegetable crop that occurred in all sampled kebeles was *Brassica carinata*. The majority of food crops were herbs: 35 (41%), followed by shrubs: 26 (30.6%), trees: 18 (21%), and climbers: 6 (7%).

### Medicinal plants

A total of 83 plant species with medicinal values were recorded, and this accounted for 35% of the total plant species documented. Species of the families Asteraceae, Rosaceae, Fabaceae, Rutaceae, Lamiaceae, Cucurbitaceae, Solanaceae, and Poaceae were the most used for traditional remedy preparation, representing 38.6% of all medicinal plants. The majority of medicinal plants were shrubs (31, 37.7%), followed by herbs (21, 25.3%), trees (23, 27.7%), and climbers (8, 9.6%). In all the studied sites, *Justicia schimperiana*, *Ruta chalepensis*, *Zehneria scabra*, *Eucalyptus globulus*, and *Vernonia amygdalina* were common medicinal plants.

### Environmental uses

Environmentally useful plants were distinguished for their uses as fences, hedges, shade, and ornamental uses. A total of 140 (59%) plant species under 66 families were recorded for various environmental uses such as living fences, hedges, shade plants, and ornamental value. Fabaceae contained the largest number of plant species (15 species) for environmental uses, followed by Asteraceae (10 species), Rosaceae (8 species), Moraceae and Poaceae (6 species each), Apocynaceae, Arecaceae, Capparidaceae, Commelinaceae, Cupressaceae, Loganiaceae, Malvaceae, Rubiaceae, Rutaceae, Sapindaceae, Solanaceae, Tiliaceae, and Verbanaceae that had 2 species each. The larger number of plant species used for environmental purposes was trees 54 (38.6%), followed by shrubs 49 (35%), herbs 30 (21.4%), and climbers 7 (5%). 96.4% of environmentally useful plants were perennials and 3.6% were annuals.

### Live fence

Live fence species constituted 112 plant species, accounting for 47% of all the plant species documented and 85.7% of the environmentally useful plants recorded. In all studied kebeles, *Justicia schimperiana*, *Eucalyptus globulus*, and *Vernonia amygdalina* were common live fence plants.

### Ornamental plants

Ornamental plant species covered 21% (50 species) of the total plant species recorded and 35.7% of the environmentally useful plants. *Cupressus lusitanica* was commonly reported among all the sample kebeles, followed by *Rosa richardii* and *Dahlia pinnata*. Most of the ornamental plants were recorded from the Wenka kebele.

### Materials

Plants in this category were those reported for their uses in construction materials, agricultural tools, roof thatch, a variety of culturally valuable instruments, and rope making. A total of 57 plant species (24%) were recorded. The commonly reported plants cited were *Eucalyptus globulus*, *Eucalyptus camaldulensis*, and *Eucalyptus saligna*.

### Social use

The social use plants were the ones reported for their ritual and religious uses, stimulant drugs, smoking, cosmetics, and baking agents. A total of 49 (20.6%) plant species under 25 families were identified for their social uses. The highest number of socially useful plants was found in the family Poaceae (7 species), followed by Cyperaceae (6 species), and Asteraceae and Lamiaceae each having 5. The growth form of social use plants indicated the dominance of herbs with 25 (50%), followed by shrubs with 13 (26%), trees with 11 (22%), and climbers with 1 (2%).

### Fragrant and stimulant plants

Individuals utilize fragrant plants to change the scent of the encompassing fabric, which can be utilized as a family utensil or can be utilized as an input for commercially made fragrances. In total, 15 (6.3%) fragrant plant species were identified in the study kebeles. And among the fragrant plants, *Ruta chalepensis* and *Olea europaea* subsp. *cuspidata* were common in all kebeles. Three (1.26%) stimulant plant species were recorded in the study area. *Coffea arabica* was found in all the study kebeles, and *Catha edulis* was widely dispersed.

### Plants used for cooking

Plants utilized for bread and potato cooking were *Ensete ventricosum* and *Galium aparinoides* which were found to be 0.84%*.*

### Fodder

A total of 39 (16.3%) plant species belonging to 19 families were found as feed for cattle, sheep, and donkeys. The Poaceae family had 11 plant species: Fabaceae and Cyperaceae, with 5 and 4 plant species, respectively. The most prevalent fodder plants were *Snowdenia polystachya*, *Cynodon dactylon*, *Zea mays*, *Vernonia amygdalina*, *Vernonia myriantha*, and *Malva verticillate*.

### Fuel

In the study area, there were 60 fuel plant species belonging to 30 botanical families, accounting for 25% of the total. The bulk of plant species used for fuel was from the families Fabaceae, Moraceae, and Myrtaceae which accounted for 16.7% of fuel plant species, whereas Anacardiaceae, Asteraceae, Poaceae, and Rosaceae accounted for 20% of the fuel plant species in the study area.

### Poison

Only 1 (0.42%) of plant species was recorded under this category.

### Direct matrix ranking

Direct matrix ranking results indicated that *Cordia africana* ranked as the most widely harvested plant for its multipurpose uses, followed by *Ficus sur* (Table 4).

**Table 4:** Average direct matrix ranking result of fifteen multipurpose species

Species	Use diversity								
	Food	Fodder	Environmental uses	Medicine	Materials	Fuel	Social uses	Total	Rank
*Vernonia amygdalina* Del	0	5	5	5	2	4	5	26	3
*Cordia africana* Lam	4	4	4	4	5	5	4	30	1
*Cupressus lusitanica* Mill	3	3	4	0	5	4	0	19	12
*Dracaena steudneri* Engl	0	4	3	5	0	0	5	17	14
*Ficus sur*Forssk	5	4	5	4	3	4	3	28	2
*Ficus vasta* Forssk	4	4	5	0	4	4	4	25	4
*Acacia abyssinica* Hochst. ex	3	4	4	4	4	4	0	23	6
*Albizia schimperiana* Oliv	0	4	3	4	5	4	4	24	4
*Myrica salicifolia* Hochst. ex A. Rich	0	5	5	4	4	4	0	22	7
*Syzygium guineense* (Willd.) DC. subsp. *Guineense*	5	4	3	0	4	4	0	20	9
*Olea europaea* L. subsp.*cuspidata* (Wall. exG. Don) Cif	0	0	3	5	5	3	5	21	8
*Prunus africana* (Hook.f.) Kalkm	0	0	3	0	5	5	5	18	13
*Salix subserrata* Willd	0	4	3	0	4	4	5	20	9
*Grewia ferruginea* Hochst. exA. Rich	4	5	3	4	0	4	0	20	9
*Celtis africana* Burm. f	0	4	2	0	4	4	0	14	15
Total	31	53	53	39	54	54	40		
Rank	7	3	3	5	1	1	5		

The results revealed that these multipurpose cultural plant species are currently used more for making different materials and fuel (firewood and charcoal) than for other cultural functions.

### Cultural importance

See Table 5.

**Table 5:** Cultural importance index (CI) of the 15 most multipurpose species of the Gozamin District, with the CI component of each use category

Species	Food	Fodder	Environmental use	Medicine	Material	Fuel	Social use	Total CI
*Vernonia amygdalina* Del		0.36	0.39	0.39	0.25	0.39	0.39	2.17
*Cordia africana* Lam	0.35	0.35	0.35	0.25	0.35	0.35	0.22	2.23
*Cupressus lusitanica* Mill	0.27	0.14	0.27		0.27	0.27		1.24
*Dracaena steudneri* Engl		0.11	0.16	0.16			0.01	0.44
*Ficus sur* Forssk	0.10	0.10	0.10	0.08	0.10	0.10	0.09	0.68
*Ficus vasta* Forssk	0.02	0.02	0.02		0.02	0.02	0.02	0.10
*Acacia abyssinica* Hochst.ex	0.08	0.15	0.15	0.07	0.10	0.15		0.70
*Albizia schimperiana* Oliv		0.12	0.14	0.11	0.14	0.14	0.11	0.76
*Myrica salicifolia* Hochst. ex A. Rich		0.02	0.02	0.02	0.02	0.02		0.10
*Syzygium guineense* (Willd.) DC. subsp. Guineense	0.03	0.03	0.03		0.02	0.02		0.11
*Olea europaea* L. subsp. *cuspidata* (Wall. exG.Don)Cif			0.30	0.28	0.30	0.14	0.30	1.32
*Prunus africana* (Hook.f.) Kalkm			0.09		0.09	0.09	0.09	0.35
*Salix subserrata* Willd		0.03	0.03		0.03	0.03	0.03	0.17
*Grewia ferruginea* Hochst. exA. Rich	0.01	0.01	0.01	0.01		0.01		0.06
*Celtis africana* Burm. f		0.25	0.35		0.35	0.35		1.30

The highest CI = 2.23 was recorded for *Cordia africana* followed by *Vernonia amygdalina* (CI = 2.17)

### Multipurpose plant species comparison under different indices

Table 6 compares the three indices described in the indicating species ranking based on each index and the three main values of the study, namely frequency of citation (FC), number of use reports (UR), and number of uses (NU) for each species. FC simply takes into account the existence of culturally beneficial plants in the garden (number of people that mention them as useful in their garden). The other indices considered the multitude of uses as well (number of use categories mentioned for a species). The highest cultural importance (CI = 2.23) and cultural value (CV = 0.68) were recorded for *Cordia africana*. The largest frequency of citation (REF = 0.39) was recorded for *Vernonia amygdalina.*

**Table 6:** Evaluation of the first 15 multipurpose useful plants of the study area, using three quantitative indices

Species	Basic value			Index			Ranking		
	Nu	FC	UR	CI	CV	RFC	CI	CV	RFC
*Vernonia amygdalina* Del	6	139	776	2.1737	0.6347	0.3894	2	2	1
*Cordia africana* Lam	7	125	795	2.2269	0.6823	0.3501	1	1	2
*Cupressus lusitanica* Mill	5	98	442	1.2381	0.2124	0.2745	5	5	5
*Dracaena steudneri* Engl	4	56	157	0.4398	0.0345	0.1569	9	9	6
*Ficus sur* Forssk	7	36	241	0.6751	0.0596	0.1008	8	8	9
*Ficus vasta* Forssk	6	6	36	0.1008	0.0013	0.0168	13	13	14
*Acacia abyssinica* Hochst. ex	6	54	249	0.6975	0.0791	0.1513	7	6	7
*Albizia schimperiana* Oliv	6	49	270	0.7563	0.0779	0.1373	6	7	8
*Myrica salicifolia Hochst. ex A. Rich*	5	7	35	0.0980	0.0012	0.0196	14	14	13
*Syzygium guineense* (Willd.) DC. subsp. *Guineense*	5	9	41	0.1148	0.0018	0.0252	12	12	12
*Olea europaea* L. subsp.*cuspidata* (Wall. exG.Don)Cif	5	107	471	1.3193	0.2471	0.2997	3	3	4
*Prunus africana* (Hook.f.) Kalkm	4	31	124	0.3473	0.0151	0.0868	10	10	10
*Salix subserrata* Willd	5	12	60	0.1681	0.0035	0.0336	11	11	11
*Grewia ferruginea* Hochst. exA. Rich	5	4	20	0.0560	0.0004	0.0112	15	15	15
*Celtis africana* Burm. f	4	125	465	1.3025	0.2280	0.3501	4	4	2

CI = cultural importance, RFC = relative frequency of citation, CV = cultural value, FC = frequency of citation, UR = number of use reports, NU = number of uses

### The similarity of homegardens among Kebeles

For all the plants collected across the 357 homegardens, Sorenson's index was calculated. Table 7 shows the species composition similarity index values across homegardens in the twelve kebeles. The highest similarity index was recorded for Yeboargina and Kebi, Yeboarginaand Yetegan, indicating that they shared 60% of the plant species. Chimit and Graram, on the other hand, had the lowest similarity values, that is they shared only 25% of the plant species. The similarity index was larger than 0.5 in 36% of the cases and less than 0.5 in 64% of the cases.

**Table 7:** Level of similarity index in composition of plant species among Kebeles

Study kebeles	Wenka	Addis enagulit	Yeboargina	Kebi	Desa Enese	Aba Libanos	Denba	Chimit	Enerata	Graram	Yebokla	Yetegan
Wenka	1											
Addisenagulit	0.46	1										
Yeboargina	0.44	0.52	1									
Kebi	0.41	0.51	0.6	1								
Desa Enese	0.35	0.48	0.51	0.45	1							
Aba Libanos	0.42	0.43	0.42	0.39	0.43	1						
Denba	0.38	0.45	0.51	0.49	0.53	0.55	1					
Chimit	0.26	0.38	0.4	0.4	0.46	0.33	0.48	1				
Enerata	0.45	0.58	0.5	0.5	0.46	0.43	0.42	0.35	1			
Graram	0.28	0.35	0.37	0.41	0.29	0.32	0.3	0.25	0.42	1		
Yebokla	0.36	0.46	0.45	0.43	0.38	0.35	0.37	0.33	0.44	0.39	1	
Yetegan	0.43	0.58	0.6	0.52	0.5	0.46	0.51	0.39	0.52	0.37	0.43	1

## Discussion

### Composition of homegarden plants

Homegardens are crucial in the conservation of beneficial plant species since they contain numerous species that are often absent or disappearing from other production systems. A total of 238 culturally important plant species were recorded, and this is supported by [12]. The composition and use of homegarden plants were studied in some parts of Northwest Ethiopia. For example, in the Jabithenan District, a total of 69 species from 40 families were reported [41], from the Bulen District, 22 plant species from 15 families were recorded [29], and from Southern Tigray, Northern Ethiopia, 32 plant species from 20 families were recorded [17]. Our findings indicated that a high number of culturally important plant species (238) were recorded in the homegardens of the Gozamin District. This relates to the rich culture of people in the area in conserving useful species at their gardens. About 110 (46%) plant species of the culturally important species that were conserved in the homegarden are emerged from the wild sources. The presence of 238 culturally important species at the homegarden of the study area showed that the homegardens are sites of in situ conservation; the finding conforms with that of [18, 19].

The size of homegardens used for plantation varies from 0.015 to 0.5 ha indicating variations in homegarden sizes harboring culturally important plants which are core sources of subsistence. Earlier reports also indicate the presence of size differences in homegardens of the Northwest Ethiopia. For example, the home garden size in Jabithenan District ranges from 0.05 to 0.5 ha [42], and it ranges from 0.031 to 0.75 ha in Bulen District (30). Of the total 357 homegardens (8.98hactares) sampled, 238 plant species were recorded. As shown in Fig. 3, the number of species grows as the size of homegardens increases but falls in homegardens with three plots. This is not surprising given that the number of home gardens that took three plots from 357 home gardens was 52 (14.6%) of the samples, and while the number of home gardens may affect the number of species, in other cases when the size increases, the number of species increases, and this is true in all the three agroclimatic zones. A comparable study conducted in Ethiopia's Oromia Region's Sebeta-Awas District [42] reported that as the size of a homegarden increases, so does the diversity of plant species. The plants recorded are trees, shrubs, herbs, and climbers. Of these, herbs were the most dominant species (39%). Reports of Mekonen et al. [42] and Regassa [43] also go in line with the findings of this study, whereas Mengitu and Fitamo [44] pointed out that trees were the dominating species in the Dilla Zuriya District. The Poaceae, Fabaceae, and Asteraceae families had the most species in the study area. This might be owing to the dominance of the Fabaceae, Asteraceae, and Poaceae families in Ethiopian and Eritrean flora, as mentioned in [45–48]. These homegarden areas, where many plant species live, have cultural importance and hence play an important role in the conservation of threatened species.

### Diversity of homegarden plant species in three agroclimatic zones

Among the three agroclimatic zones where the homegarden plants were recorded, *Woina Dega* comprised the highest number of plant species (203) accounting for 85.3% of the total number of species in the samples, followed by the *Kolla* agroclimatic zone (130) (Table 2). The result indicated that *Woina Dega* agroclimatic zone was home to a wide variety of plant species. This is substantially owing to the fact that the *Woina Dega* agroclimatic zone might be ideal for the growth of many plant species. Abebe [25] also showed that the agroclimatic zones of Woina Dega have a high potential for perennial cropping. It also had the highest sample size (53.5%). Although the diversity of home garden plant species was lowest in *Dega* agroclimatic zone (88), it had the highest evenness, highlighting the importance of this agroclimatic zone. We discovered an *Ensete ventricosum* plantation (enset variety used as food) in the *Dega* agroclimatic zone; it was native to Ethiopia's south and southwestern regions, where it is now commonly farmed [25]. This is a promising start to conserve *Ensete ventricosum* and secure food availability in the research area, where the livelihood of the people mainly relies on cereal crops.

### Homegarden plant species composition similarities

The highest similarity index was found between Yeboargina and Kebi, and Yboargena and Ytegan, indicating that they shared 60% of the plant species. This might be because they are found in a similar agroecological zone. On the other hand, Chimit and Graram had the lowest similarity values, meaning that they shared only 25% of the plant species. The reason could be the agroecology difference between them. The similarity index for 64% of the locations (Kebeles) investigated was less than 0.5, suggesting that there was less similarity to high species diversity in the area. This might be due to differences in agroecological conditions among Kebeles, as evidenced by Ertiro et al. [49] who demonstrated that agroecology has an impact on variation.

### Plant use diversity

From sampled homegardens, plant species used for food, fodder, environmental uses, fuels, medicines, materials, socially useful plants, and rat poison were recorded. This finding agrees with Nair et al. [28], who showed gardens' enormous species diversity, which consists of food crops, medicinal plants, ornamentals, fruit trees, multipurpose trees, and fodder species, supporting a variety of ecosystem services. From the use composition of homegarden plants in the study area, the highest record of use composition was food, which means homegardens primary use value was food, secondly medicine for humans and animals, followed by social uses (ritual and religious uses, stimulant drugs, cosmetics, and baking agents) (Fig. 7). The same is true in the three agroclimatic zones; the first and second most important use diversity were recorded for food and medicinal uses, but the third cultural use in *Dega* and *Kolla* agroecology was environmental, and the third cultural use in *Woina Dega* agroecology was social use (Fig. 6). Plant composition varies between agroecological zones as a result of changes in niche quality and the adaptation of environmental gradients such as precipitation, temperature, and soil fertility, as agroecology variation is mostly related to altitudinal gradient. According to [26], elevation has an impact on species composition. The cultural value difference in different agroecological zones relates to species availability (which is based on variations in environmental factors), and people’s preferences corresponding to their different living trends. This finding is consistent with Song et al. [50], who found that gardeners' profiles were strongly correlated with community food provisioning services and may have social and environmental benefits. That is, plant species that were used for food, medicine, and social uses have been conserved more than others in the study area. This also shows that culture (cultural use) has a high value for conserving plant species. This finding agrees with [37].

### The cultural importance of homegarden plants

**Fig. 8:**
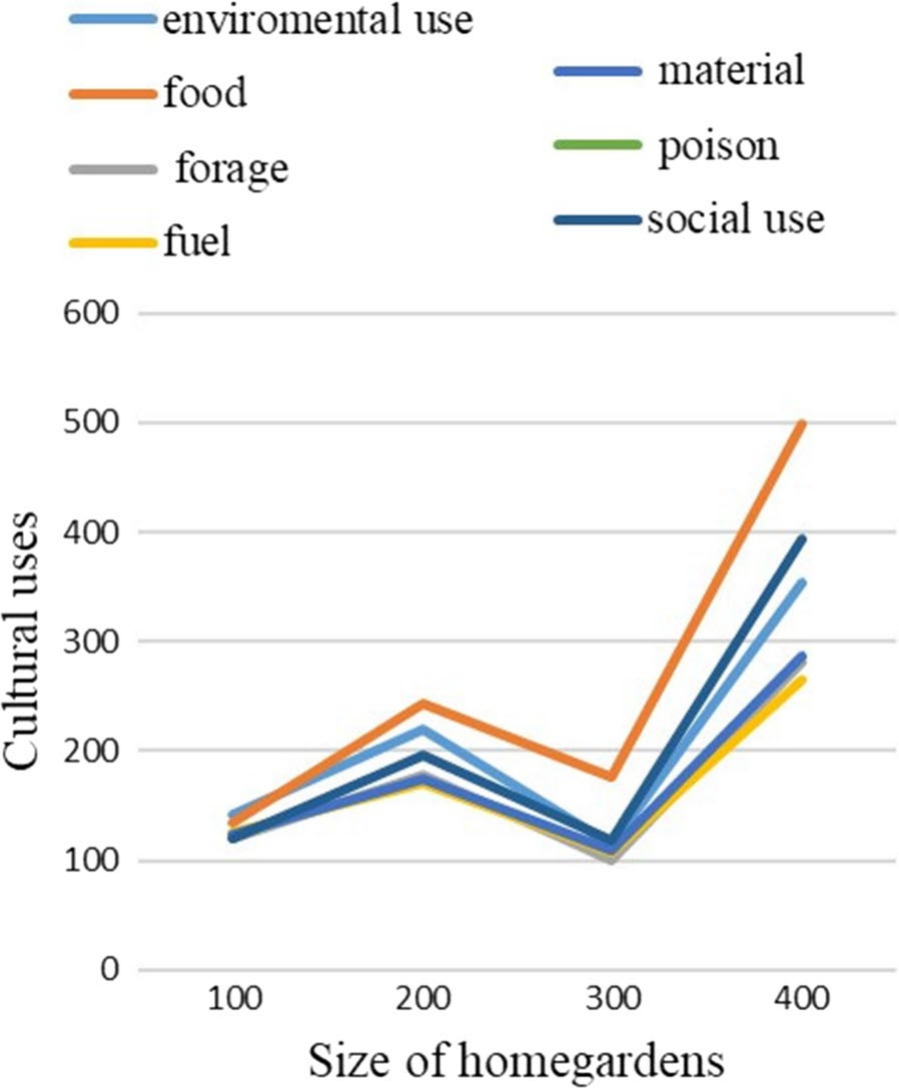
Size of homegarden and cultural use of species in Gozamin District

People in the study area have designed diverse and adaptable homegardens that grow a variety of important livelihood crops. The homegardens remarkably possess plant species used for human and veterinary medicines, livestock feed, and plant species used to make different materials (such as handicrafts, construction materials, agricultural tools, roof thatch, instruments, toothbrushes, rope, mortar, and pestle), fuel, social utility (ritual and religious uses, stimulant drugs, smoking, cosmetics, and baking agents), environmental uses (such as live fence, dry fence, hedges, shade, ornamental and soil improvement), and this trend is also true in other studies [40]. The community members in the area have cultural knowledge in crop selection and information sharing with culturally knowledgeable people in the study area, which co-adapts agroecology and provides multiple benefits [51]. As shown in Figs. 8 and 3, the size of the homegarden increases the number of species and the use diversity, but not in the third instance (which took 3 plots). This scenario is similar to the previous one, in that fewer homegardens were collected and fewer species were documented, but in other cases, the number of species and use diversity of homegardens increased as homegarden size increased. Moore et al. [52] and Manne [53] also showed that species diversity and cultural diversity were intimately correlated. Gardens are located around water-abundant areas (rivers, ponds, and water wells). Amede and Taye [54] also showed that surface irrigation water (springs and rivers) had become a key motivator for cultivating fruits and vegetables in Ethiopian homegardens.

Homegardens in the study area were mostly live-fenced or semi-fenced areas. Trees, shrubs, herbs, and climbers were used as live fences, serving as a barrier to keep predators away from fruits, vegetables, spices, and other items. This culture in the study site keeps diverse plant species around people’s homes and gardens and allows for in situ conservation of species which are still in use for fences and other cultural values. The present study identified that the majority of the homegardens analyzed were encircled by a live fence. This was also shown by other studies [16, 29]. In general, environmentally useful plants such as fences, shade, and ornamental plants cover 140 (58.6%) plant species under 66 families. This was supported by Kumar and Nair [4], who showed that homegardens were praised for preserving biodiversity and preventing environmental degradation.

Environmental variables and dietary preferences, as well as socioeconomic and commercial needs, are all reported for their influence on the distribution of species in homegardens [55]. As observed in the current study area, the number of plant species varied between kebeles. Since there was irrigation water, and Wenka is close to the main town of Debre Markos, it had a significant number of home garden species (130 species in 58 families). The site supplied food crops to Debre Markos town, primarily fruits, and vegetables, and this encouraged farmers to sell their homegrown produce in areas with strong market access [15, 25].

In the study area, homegardening provides subsistence food production and family food security by producing vegetables, fruits, cereals, spices, and beverages. Other authors [26, 42–44, 56, 57] have also reported on the widespread use and service of homegardens. Fruits were the most commonly utilized plant parts from food crops in the studied homegardens. The result goes in line with other reports [43, 44, 58].

Information about medicinal plants (passed from grandparents) was exchanged among indigenous peoples and neighbors and played its role in medicinal plants conservation. Exchange of plant resources and cultural information among communities has been reported as a useful tool to promote conservation [59]. A total of 83 therapeutic plants were recorded at homegardens of the study site. This demonstrates that gardening has placed a greater emphasis on human health and well-being [60], the main healthcare provider [61]. *Dracaena steudneri* was found with medicinal properties for treating livestock ailments. It was also cited for treating bad spirits, besides its decorative nature, fodder, shade plant, fence, and other cultures, and hence was common within the study area.

Some socially useful plants such as *Ficus sur*, *Ficus vasta*, and *Prunus africana* are used by local people in the area for different rituals and religious uses; *Prunus africana,* for example, is used for decorating wedding ceremonies in the area and hence conserved by local communities. Plants used for religious ceremonies, stimulants, smoking, and baking account for 50 (21%) of the species identified. This proves that cultural value has become the center point for preserving plants [42, 61, 62] and has also reported similar records. *Coffea arabica*, which was also used in households and for the market, was discovered in all the study kebeles, and *Catha edulis*, which was also utilized for the market to generate income, was also detected in most study sites (kebeles). This was also shown in other research findings [15, 29]. Also, Mellisse et al. [63] recently reported that there has been a dramatic movement away from conventional family gardens toward cash crop *Catha edulis*-based systems, especially in locations near to marketplaces. A total of 2 (0.84%) plants were found to be utilized in the preparation of bread and potatoes. *Ensete ventricosum*, which was used for baking bread, potting injera, and malting malt, was described as having a nutraceutical nature only [42, 64–66]; *Galium aparinoides* was a weed that was commonly used to cook potatoes since it cooked them quickly and had a wonderful flavor; however, Mekonen et al. [42] classified it as a weed that affects the variety and productivity of homegarden plants. Local communities also conserve fodder plants around their homegardens, mostly for fattening cattle and sheep. *Snowdenia polystachya*, which is a perennial plant species, was the highest fodder plant recorded, followed by *Cenchrus ciliaris* and *Cynodon dactylon*. Likewise, the root multifunctional plants that have high usable values are threatened since accessed by the local community for their multiple uses, even though they are the foundations of most tropical backyard gardens [28]. Multipurpose cultural plant species are threatened primarily for the production of various materials and fuel (firewood and charcoal) rather than for other cultural activities. The same was true in North Shewa Zone, Amhara Region, Ethiopia [67]. The direct matrix index revealed that *Cordia africana*, a multifunctional, culturally important plant in the area, was the most extremely endangered species, followed by *Ficus sur*. *Cordia africana* is exploited more for construction. People are also using *Cordia africana* to make different materials. *Cupressus lusitanica* was a common plant in all sampled kebeles with multipurpose functions including for purposes of ornamental use, construction and building, as gum used in food, forage, fence, and fuel, and this report matches with that of Mekonen et al. [42]. Similarly, by the cultural importance index, the first multifunctional plant, *Cordia africana* (FC = 125), is the most culturally significant species. It has a value of 2.23 on the CI index. This plant species is mostly used for food, fodder, environmental use, material and fuel (CI = 0.35), medicinal use (CI = 0.25), and social use (CI = 0.22), as shown in Table 5, contradicting the findings observed in the direct matrix ranking; the cultural value index considers not only the prevalence of use (number of informants) for each species, but also its versatility, i.e., the variety of its uses. The maximum value of the index is the entire number of various use categories in their garden (NC). That is, the CI index considers the species UR and FC (*Cordia africana*) has the first UR (795) and second FC value (125)) and also reflects the distribution of species because it is the total of the proportion of informants who mention each species' use in their garden. *Vernonia amygdalina* (CI = 2.17) is the second most culturally important species in the ranking. As shown in the figure for CI index components in Table 5, the most common uses from the seven uses categories are medicine, fuel, and social use (CI = 0.39), followed by feeding (CI = 0.36) and material use (CI = 0.25). *Olea europaea* subsp. *cuspidata* (CI = 1.32) was the third culturally significant species, and it was employed for the environment, material and social usage (CI = 0.3), medicinal usage (CI = 0.28), and fuel (CI = 0.14). Also, people in the study area believed that the fumigation of *Olea europaea* subsp. *Cuspidata* in the house using leaves and stems removed bad spirits and bad odors from the house and were also used to fumigate milk, Tela, and Tej (local beverages) pots [68]. This finding indicates the presence of high level of agreement on cultural relevance of species, and the sharing of common knowledge on useful species by homegarden owners. This finding is supported with that of [23, 38]. In this study, the multipurpose species that have been identified in a wide number of home gardens (greater FC) were given first place for conservation and cultural use. This was also true for Tardío and Pardo-de-Santayana [38].

There are some variations in the ranks of species analyzed using various indicators as shown in Table 5. Although the third, tenth, twelfth, thirteenth, and fourteenth species have the same rank in all three of the indices, The CI and CVI indices rank *Cordia africana* first and foremost because these two indices place greater emphasis on the multiplicity of uses, and the species was cited in a greater number of use categories (NU = 7). *Vernonia amygdalina* has a higher IFC since it predominates in homegardens and is present in a greater number of them. Preserving cultural importance entails expanding understanding and safeguarding culturally important plant species. Our results suggest considering cultural use and the cultural importance of floristic composition before designing biodiversity conservation in agroecosystems.

## Conclusion

In the present study, we recorded 84 food plant species. Homegardening enables subsistence food production and secures family food security by producing vegetables, fruits, cereals, spices, and beverages. In addition, homegardens in the study area are used as multifunctional for communities. Culturally important plant species have high value in terms of family household income production, therapeutic, decorative, and other non-food ways of life, and their cultural value may also be a key figure in biodiversity preservation. Food security and biodiversity conservation are continuously supported by tribal populations' cultural knowledge connected with their homegardens. Because there were numerous homegarden plant species within the study area and the area had less forest cover, legitimate homegarden management is the most vital way to preserve biodiversity and contribute to ecosystem services in the considered area. In three agroclimatic zones, there is a variation in species composition and use diversity. The multipurpose species with the highest cultural relevance were prioritized for conservation. As the garden increases in size, so does the variety of plants and uses. Conserving cultural use, the cultural importance of the species is central to conserving plant species in the area. Our findings suggest that conservation plans should take into account the determinants of floristic composition as well as the relationships between floristic composition and cultural importance.

## Data Availability

All data analyzed during this study are included in this published article.

## Appendix 1: The distribution of the interviewees in relation to community, gender, and age

**Table:**

Locallity	Total	20–40	41–60	61–86
Abalibanos				
M	21	4	13	4
F	9	3	4	2
Addisenagulit				
M	28	8	14	6
F	2	–	1	1
Chimit				
M	21	7	6	8
F	6	3	1	2
Denba				
M	18	1	10	7
F	12	4	5	3
Desaenesa				
M	25	7	10	8
F	5	1	1	3
Enerata				
M	24	6	13	5
F	6	2	2	2
Geraram				
M	13	2	7	4
F	17	11	4	2
Kebi				
M	18	6	6	6
F	12	2	6	4
Wonka				
M	15	4	6	5
F	15	5	6	4
Yeboargna				
M	24	7	3	14
F	6	2	3	1
Yebokla				
M	24	8	12	4
F	6	2	3	1
Yeregan				
M	24	6	13	5
F	6	2	4	–

## Appendix 2: Test for normal distribution

**Table:**

	*Dega*	*Kolla*	*Woina Dega*
*N*	237	237	237
Shapiro–Wilk W	0.4795	0.4878	0.6088
*p*(normal)	7.839E−26	1.161E−25	6.786E−23
Anderson–Darling A	46.41	43.38	31.41
*p*(normal)	4.283E−98	9.14E−93	2.808E−70
*p*(Monte Carlo)	0.0001	0.0001	0.0001
Lilliefors L	0.3385	0.3306	0.2833
p(normal)	0.0001	0.0001	0.0001
*p*(Monte Carlo)	0.0001	0.0001	0.0001
Jarque–Bera JB	2848	2539	1342
*p*(normal)	0	0	4.112E−292
*p*(Monte Carlo)	0.0001	0.0001	0.0001
